# Formulation Development and Evaluation of Fast Disintegrating Tablets of Salbutamol Sulphate for Respiratory Disorders

**DOI:** 10.1155/2013/674507

**Published:** 2013-07-15

**Authors:** Deepak Sharma

**Affiliations:** CT Institute of Pharmaceutical Sciences, Shahpur, PO Udopur, Near Lambra, Jalandhar, Punjab 144020, India

## Abstract

Recent developments in fast disintegrating tablets have brought convenience in dosing to pediatric and elderly patients who have trouble in swallowing tablets. The objective of the present study was to prepare the fast disintegrating tablet of salbutamol sulphate for respiratory disorders for pediatrics. As precision of dosing and patient's compliance become important prerequisites for a long-term treatment, there is a need to develop a formulation for this drug which overcomes problems such as difficulty in swallowing, inconvenience in administration while travelling, and patient's acceptability. Hence, the present investigation were undertaken with a view to develop a fast disintegrating tablet of salbutamol sulphate which offers a new range of products having desired characteristics and intended benefits. Superdisintegrants such as sodium starch glycolate was optimized. Different binders were optimized along with optimized superdisintegrant concentration. The tablets were prepared by direct compression technique. The tablets were evaluated for hardness, friability, weight variation, wetting time, disintegration time, and uniformity of content. Optimized formulation was evaluated by in vitro dissolution test, drug-excipient compatibility, and accelerated stability study. It was concluded that fast disintegrating tablets of salbutamol sulphate were formulated successfully with desired characteristics which disintegrated rapidly; provided rapid onset of action; and enhanced the patient convenience and compliance.

## 1. Introduction

Despite tremendous innovations in drug delivery, the oral route remains the preferred route for administration of therapeutic agents because of accurate dosage, low cost therapy, self medication, noninvasive method, and ease of administration leading to high level of patient compliance [1]. The most popular dosage forms are conventional tablets and hard gelatin capsules. One important drawback of such dosage forms is “dysphagia” or difficulty in swallowing for many patients; almost 50% of the population is affected by such problem. Hence, patients do not comply with prescription, which results in high incidence of noncompliance and ineffective therapy [2].Recently, fast disintegrating drug delivery systems have started gaining popularity and acceptance as new drug delivery systems, because they are easy to administer and lead to better patient compliance [3]. In some cases such as motion sickness, sudden episodes of *allergic attacks or coughing,* and unavailability of water, swallowing conventional tablets may be difficult. Particularly the difficulty is experienced by pediatric and geriatric patients. To overcome such problems, fast disintegrating tablets or orally disintegrating tablets have emerged as an alternative dosage form [4]. Recent advances in novel drug delivery systems (NDDS) aim for enhancing the safety of a drug molecule while maintaining its therapeutic efficacy so as to achieve better patient compliance [5].

US Food and Drug Administration Center for Drug Evaluation and Research (CDER) defines, in the “Orange Book,” an ODT as “a solid dosage form containing medicinal substances, which disintegrates rapidly, usually within a matter of seconds, when placed upon the tongue.” European Pharmacopoeia described ODTs as “uncoated tablets intended to be placed in the mouth where they disperse rapidly before being swallowed” and as tablets which should disintegrate within 3 minutes [6]. Fast disintegrating tablets (FDTs) are also known as “fast dissolving,” “mouth dissolving,” “rapid dissolve,” “quick disintegrating,” “orally disintegrating,” “rapimelt,” “fast melts,” “orodispersible,” “melt in mouth,” “quick dissolving,” “porous tablets,” “EFVDAS,” or “effervescent drug absorption system” [7].

The bioavailability of drugs may be increased due to absorption of drug in oral cavity and also due to pregastric absorption of saliva containing dispersed drugs that pass down into the stomach. Moreover, the amount of drug that is subjected to first-pass metabolism is reduced as compared to standard tablet [8]. The target populations for these new fast-dissolving/disintegrating dosage forms have generally been pediatric, geriatric, and bedridden or mentally disabled patients. Patients with *diarrhea, persistent nausea, *or *vomiting,* who are traveling, or who have little or no access to water are also good candidates for FDTs [9].

Formulation of the drug chosen for the treatment of asthmatic cough and other respiratory disorders is available in market in conventional tablet and liquid dosage forms. Liquid dosage forms are having their own limitation from stability and dose measurement perspectives. Tablets to be swallowed are resisted by children patients, and patient compliance is an issue with such dosage forms. Hence, they do not comply with the prescription, which results in high incidence of noncompliance and ineffective therapy. Fast disintegrating tablet dosage form offers a mean of delivering drugs to children and other patients who have faced difficulty in swallowing tablets and for those suffering from diarrhea. Salbutamol sulphate is a short-acting ß2-adrenergic receptor agonist used for the relief of bronchospasm in conditions such as asthma and (chronic obstructive pulmonary disease COPD) [10, 11]. Due to sore throat conditions, the patient experiences difficulty in swallowing a tablet type of dosage form. Thus, fast disintegrating tablets would serve as an ideal dosage form for pediatric patients who find it difficult to swallow the tablets [11]. Hence, an attempt was made for preparation of fast disintegrating tablet of salbutamol sulphate with an aim of improving/enhancing patient convenience and compliance, reducing the lag time and providing faster onset of action to relieve the respiratory disorders immediately. 

## 2. Materials and Methods

### 2.1. Materials

Salbutamol sulphate was received as gift sample from Trojan Pharma, Baddi, India. Microcrystalline cellulose (Avicel PH-102) was obtained as gift sample from NB Entrepreneurs, Nagpur, India. Sodium starch glycolate (Primogel, Explotab) and directly compressible Mannitol (D-Mannitol) were purchased from Qualikems Fine Chem Pvt. Ltd. Sodium stearyl fumarate was purchased from Himedia. Sodium saccharin was purchased from Loba Chemie, Mumbai, and talc from Nice Chemicals Private Limited, Hyderabad, India. All other chemicals and reagents that were of analytical grade were used.

### 2.2. Methods

#### 2.2.1. Selection of Excipients and Optimization of Their Concentration

The most important parameter that needs to be optimized in the development of fast disintegrating tablets is the disintegration time. Fast disintegrating tablets were prepared firstly using different excipients (binders and superdisintegrants) and then evaluated for various parameters like friability, hardness, and disintegration time to select the best combination for formulation of fast disintegrating tablets. The combination with lowest disintegration time, optimum hardness, and friability was selected for further study. 

*(1) Optimization of Superdisintegrant Sodium Starch Glycolate (Primogel, Explotab). *For tablets and capsules which require rapid disintegration, the inclusion of the right superdisintegrant and in its optimum concentration is a prerequisite for optimal bioavailability. Superdisintegrants decrease disintegration time which in turn enhances drug dissolution rate. Thus, the proper choice of superdisintegrant and its consistency of performance are of critical importance to the formulation of rapidly disintegrating dosage forms.

Formulation F1–F6 was prepared to study the effect of type and concentration of superdisintegrants in Table 1. Tablets were prepared by direct compression technique. Weighed quantity of salbutamol sulphate with different concentrations of superdisintegrant along with excipients was mixed in geometric progression in a dry and clean mortar. Then the blend was passed through sieve number 60 for direct compression. The powder blend was then compressed into tablets using 8 mm punch in multipunch tablet compression machine (Dhiman Industries, India).

*(2) Optimization of Polyvinylpyrrolidone (PVP K-30) or Microcrystalline Cellulose (Avicel PH-102) as Binder along with Optimized Concentration of Superdisintegrant. *Tablets were prepared by direct compression technique. The composition of fast disintegrating tablet is shown in Table 2. Weighed quantity of salbutamol sulphate with optimized concentration of sodium starch glycolate along with different concentrations of binders (PVP K-30, MCC) along with excipients was mixed in geometric progression in a dry and clean mortar. Then the blend was passed through sieve number 60 for direct compression. The powder blend was then compressed into tablets using 8 mm punch in multipunch tablet compression machine (Dhiman Industries, India).

### 2.3. Final Formulation of Salbutamol Sulphate Fast Disintegrating Tablets by Direct Compression Method

Fast disintegrating tablets of salbutamol sulphate were prepared by direct compression method according to the formula given in Table 3. Weighed quantity of salbutamol sulphate along with optimized concentration of superdisintegrant and binder along with excipients was mixed in geometric progression in a dry and clean mortar. Then the blend was passed through sieve number 60 for direct compression. The powder blend was then compressed into tablets using 8 mm punch in multipunch tablet compression machine. These fabricated tablets were evaluated.

### 2.4. Evaluation Parameters

#### 2.4.1. Weight Variation

Twenty tablets were selected and weighed on digital weighting balance (Ohaus, USA), and average weight was determined. Then individual tablets were weighed, and the individual weight was compared with an average weight [12] (see Table 4).

#### 2.4.2. Thickness

Thickness of tablets was determined using vernier caliper (Indian Caliper Industries, Ambala, India). Three tablets from each batch were used, and an average value was calculated [12].

#### 2.4.3. Hardness

 The crushing strength of the tablets was measured using a Monsanto hardness tester (Perfit). Three tablets from each formulation batch were tested randomly, and the average reading was noted. The hardness is measured in kg/cm^2^ [13].

#### 2.4.4. Friability

Ten tablets were weighed and placed in a Roche friabilator (Veego, India), and the equipment was rotated at 25 rpm for 4 min. The tablets were taken out, de-dusted, and reweighed. The percentage friability of the tablets was measured as per the following formula [14]:

(1) $$\begin{array}{c} \text{Percentage}\;\text{friability}=\frac{\text{Initial}\;\text{weight}-\text{Final}\;\text{weight}}{\text{Initial}\;\text{weight}}\times 100. \end{array}$$

#### 2.4.5. In Vitro Disintegration Test

The test was carried out on 6 tablets using digital tablet disintegration tester (Veego, India). Distilled water at 37°C ± 2°C was used as a disintegration media, and the time taken for complete disintegration of the tablet with no palpable mass remaining in the apparatus was measured in seconds [15]. 

#### 2.4.6. Wetting Time

 A Petri dish containing 6 mL of distilled water was taken. A tablet containing a small quantity of amaranth color was placed on it. Time required for the upper surface of the tablet to become complete red was noted [16].

#### 2.4.7. Drug Content Uniformity

Ten tablets (200 mg) were powdered in mortar pestle, and the blend equivalent to 2 mg of salbutamol sulphate was weighed and dissolved in 100 mL of 6.8 pH phosphate buffer solutions. The solution was sonicated, filtered through whatman filter paper, and suitably diluted with 6.8 pH phosphate buffer, and the drug content was analyzed by using double beam UV spectrophotometer (UV-1800 Shimadzu) at 276 nm, respectively. Each sample was analyzed in triplicate.

#### 2.4.8. In Vitro Dissolution Study

The release of formulated FDTs was determined using USP eight-stage dissolution testing apparatus-2 (paddle method) (Lab, India). The dissolution test was performed using 500 mL of phosphate buffer solution, pH 6.8 at 37 ± 0.5°C and 50 rpm. A sample (5 mL) of the solution was withdrawn from the dissolution apparatus at specific time intervals, and the samples were replaced with fresh dissolution medium. The samples were filtered through Whatman filter paper. Absorbance of these solutions was measured at 276 nm using a double beam UV spectrophotometer (UV-1800 Shimadzu). Cumulative percentage (%) of drug release was calculated using standard plot of salbutamol sulphate [17].

#### 2.4.9. Drug-Excipient Compatibility Studies

These studies were performed in order to confirm the drug-excipient interaction. These studies mainly include FTIR spectroscopy. FTIR spectra of pure drugs and formulated FDT containing drug were recorded on FTIR spectrophotometer (Bruker, USA). The scanning range was from 4000 to 600 cm^−1^, and the resolution was 1 cm^−1^. The scans were evaluated for presence of principal peaks of drug, shifting and masking of drug peaks, and appearance of new peaks due to excipient interaction. This spectral analysis was employed to check the compatibility of drugs with the excipients used [18].

#### 2.4.10. Accelerated Stability Studies

Accelerated stability studies are conducted at temperature of 40 ± 2°C (oven) and at ambient humidity as well as at room temperature (Desiccator). The tablets were withdrawn on the 15th and 30th days and analyzed for hardness, friability, drug content uniformity, and in vitro disintegration time which are the most important parameters for fast disintegrating tablets [19].

## 3. Results and Discussion

The present investigation was undertaken to formulate and evaluate fast disintegrating tablets of salbutamol sulphate by direct compression method using sodium starch glycolate as a superdisintegrant and mannitol as directly compressible diluent, and sodium saccharin was used to enhance palatability. Avicel PH 102 was included in the formulation as a disintegrant and a binder. This grade of microcrystalline cellulose is granular in nature and thus displays excellent flow properties. To impart pleasant taste and improve mouth feel, sodium saccharin was included as sweetening agent. Sodium stearyl fumarate was employed as a lubricant instead of magnesium stearate not only because of the metallic taste of the latter, but also due to its water solubility and directly compressible features.

### 3.1. Optimization of Superdisintegrant Sodium Starch Glycolate (Primogel, Explotab)

Superdisintegrants are generally used by formulation scientists for developing FDTs or for improvement of solubility for drugs. The primary requirement for such dosage forms is quicker disintegration. The amount of superdisintegrants was optimized in the formulation of FDTs. The total 6 formulations (F1–F6) were prepared using different concentrations of sodium starch glycolate to study its effect on disintegration time. The results for optimization of superdisintegrant concentration in FDTs by direct compression method are shown in Table 5.

From the evaluation parameters, it was observed that 4% sodium starch glycolate was the optimum concentration for rapid tablet disintegration on the basis of least disintegration time observed with F3 formulation. The superdisintegrant action of SSG is resulted in hydrophilicity and swelling which in turn causes rapid disintegration. It absorbs water rapidly and swells in water to the extent of 200–300%, disintegrates rapidly. Sodium starch glycolate is used as superdisintegrant in tablet formulation at a concentration of 4–6%. Above 8% disintegration times may actually increase due to gelling and its subsequent viscosity producing effects. 

### 3.2. Optimization of Polyvinylpyrrolidone (PVP K-30) or Microcrystalline Cellulose (Avicel PH-102) as Binder along with Optimized Concentration of Superdisintegrant

The binders such as polyvinylpyrrolidone (PVP K-30) or microcrystalline cellulose were optimized with superdisintegrant concentration to further study the effect of binders on the disintegration time as well as on hardness and friability of tablets of the formulation. Total 14 formulations (F1–F14) were prepared using different concentrations of polyvinylpyrrolidone (PVP K-30) or microcrystalline cellulose to study its effect on disintegration time of formulations. The results for optimization of different binders in FDTs by direct compression method are shown in Table 6.

From the evaluation parameters, it was observed that disintegration time of the formulation was further decreased and tablet hardness and friability were within the IP limits. The least disintegration time was observed in F8 formulation, that is, 1% MCC, as compared to F2 formulation, that is, 2% PVP K-30. Water soluble materials such as PVP K-30 tend to dissolve rather than disintegrate, while insoluble materials like MCC generally produce rapidly disintegrating tablets. Due to the presence of porous morphology, liquid is drawn up or “wicked” into these pathways through capillary action and ruptures the interparticulate bonds causing the tablet to break apart. Therefore 1% microcrystalline cellulose was selected as optimum binder concentration selected for final formulation of salbutamol sulphate FDT.

### 3.3. Evaluation Parameters for Salbutamol Sulphate Fast Disintegrating Tablet

Final formulation of salbutamol sulphate FDT was tested for all the official tests of tablet and was found to be within limits as shown in Table 7. Percent weight variation was well within the acceptable limit for uncoated tablets as per Indian Pharmacopoeia. It is well known to formulation scientists that the tablets with more hardness show longer disintegration time. Since mechanical integrity is of paramount importance in successful formulation of FDTs, hence the hardness of tablets was determined. The friability of salbutamol sulphate FDT was less than 1% which is acceptable according IP criteria. The content uniformity of the prepared salbutamol sulphate FDT was complied with IP specifications. No tablet from ten tablets lies out of the range of 85–115% of the label claim. These results indicated that the dosage form had uniform distribution and proper dose of the active ingredient. The wetting time and disintegration time were practically good for formulation. According to IP, the dispersible tablet must disintegrate within 3 minutes, but the formulated FDTs have shown low DT indicating suitability of formulation for mouth dissolving tablet.

### 3.4. In Vitro Dissolution Study

**Figure 1:**
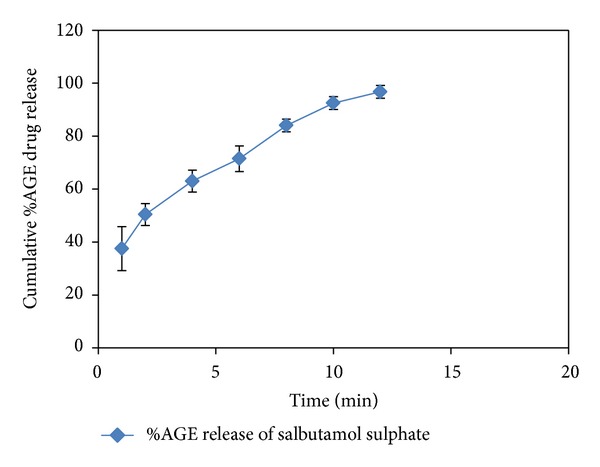
In vitro dissolution profile of salbutamol sulphate FDT.

In vitro dissolution studies showed that more than 50% of the drug was released from the formulation within 5 minutes. The rapid drug dissolution might be due to easy breakdown of particle by superdisintegrant action. From in vitro dissolution data, it was observed that 96.75 ± 2.42% of salbutamol sulphate released in 12 minutes indicates that the tablet complies as per IP specifications, that is, 85%–110% (see Figure 1). 

### 3.5. Drug-Excipient Compatibility Studies

**Figure 2:**
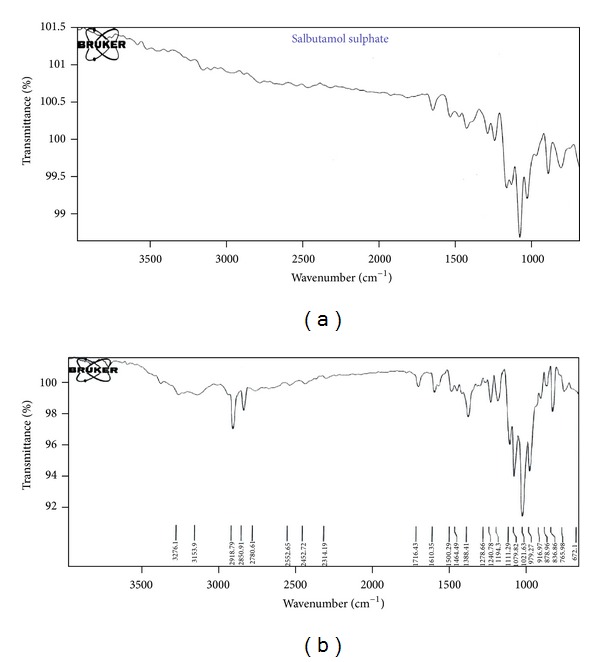
FTIR spectra of salbutamol sulphate versus salbutamol sulphate FDT.

The results obtained with IR studies showed that there was no interaction between the drug and other excipients used in the formulation. The FTIR of salbutamol sulphate has shown intense band at 1386.68 cm^−1^, 1612.60 cm^−1^, and 1386.68 cm^−1^ corresponding to the presence of functional groups such as Tri-methyl group, secondary amine group, and phenol group. The FTIR of salbutamol sulphate FDT formulation has shown intense bands at 1388.41 cm^−1^, 1610.35 cm^−1^, and 1388.41 cm^−1^ which indicates no change in the functional groups such as Tri-methyl group, secondary amine group, and phenol group and confirmed undisturbed structure of Salbutamol Sulphate, which indicates no drug-excipient interaction as shown in Figure 2.

### 3.6. Accelerated Stability Studies

In the present study, stability studies were carried out on formulated FDTs (formulated in three primary batches) wrapped in aluminium foil to prevent the formulation from exposure to light to simulate the aluminum packaging, that is, Alu Alu packing of drug products, and stored in air-tight containers which are impermeable to solid, liquid, and gases, under the following condition for one month period as prescribed by ICH guidelines for accelerated study. During the stability studies the product is exposed to normal conditions of temperature and humidity. However, the studies will take a longer time, and hence it would be convenient to carry out accelerated stability studies, where the product is stored under extreme conditions of temperature and humidity. The stability data of formulation shown in Tables 8 and 9 as given.

The result of the stability study indicated that there were not much differences observed in hardness, disintegration time, drug content uniformity, and friability before and after the storage period at room temperature and at ambient humidity, but at temperature of 40°C ± 2°C and at ambient humidity, hardness was increased with time, prolonged the DT of the tablet, and the probable reason was the loss of moisture from tablets, but in all cases, DT is within the specified IP limit (within 3 min.). This indicates that formulation is fairly stable at both storage conditions.

Statistical analysis (ANOVA) was also performed with the GraphPad InStat 3 statistical package for Windows. Stability data shown in tables for three primary batches of formulations were evaluated before and after stability testing represented mean of three or six determinations ± standard deviation (SD). Statistical significance of the differences between the evaluation parameters of three primary batches was calculated by the Tukey-Kramer multiple comparison test, and probability value of *P* smaller than 0.05 indicated a statistically significant difference.

## 4. Conclusion

Fast disintegrating tablet is a promising approach with a view of obtaining faster action of the drug and would be advantageous in comparison to currently available conventional dosage forms. The FDT dosage form had a good balance over disintegration time and mechanical strength. The prime objective of the study was to develop salbutamol sulphate fast disintegrating tablet by using commonly available excipients and conventional technology. From the study, it was concluded that by employing commonly available pharmaceutical excipients such as superdisintegrants, hydrophilic and swellable excipients, and proper filler, a fast disintegrating tablet of salbutamol sulphate can be developed which can be commercialized.

## Figures and Tables

**Table 1:** Formula for 1 tablet (200 mg) of different concentrations of sodium starch glycolate (data in mg).

Sr. no.	Ingredients	F1	F2	F3	F4	F5	F6
1	Salbutamol sulphate	2	2	2	2	2	2
2	Sodium starch glycolate	2 (1%)	4 (2%)	8 (4%)	12 (6%)	16 (8%)	20 (10%)
3	Polyvinylpyrrolidone K-30	4	4	4	4	4	4
4	Sodium stearyl fumarate	3	3	3	3	3	3
5	Talc	3	3	3	3	3	3
6	Sodium saccharin	5	5	5	5	5	5
7	Mannitol	181	179	175	171	167	163

**Table 2:** Formula for 1 tablet (200 mg) for the optimization of polyvinylpyrrolidone K-30 or microcrystalline cellulose with optimized concentration of sodium starch glycolate.

Contents	Salbutamol sulphate (mg)	SSG (mg)	PVK-30 (mg)	MCC (mg)	Sodium stearyl fumarate (mg)	Talc (mg)	Sodium saccharin (mg)	Mannitol (mg)
Formula no.								
F1	2	8	2	—	2	2	5	179
F2	2	8	4	—	2	2	5	177
F3	2	8	6	—	2	2	5	175
F4	2	8	8	—	2	2	5	173
F5	2	8	10	—	2	2	5	171
F6	2	8	12	—	2	2	5	169
F7	2	8	14		2	2	5	167
F8	2	8	—	2	2	2	5	179
F9	2	8	—	4	2	2	5	177
F10	2	8	—	6	2	2	5	175
F11	2	8	—	8	2	2	5	173
F12	2	8	—	10	2	2	5	171
F13	2	8	—	12	2	2	5	169
F14	2	8	—	14	2	2	5	167

**Table 3:** Formula of salbutamol sulphate FDT prepared by direct compression method (data in mg).

Sr. no.	Ingredients	Formula for 1 tablet (200 mg)	Formula for 110 tablets (200 mg)
1	Salbutamol sulphate	2	220
2	Sodium starch glycolate	8	880
3	Microcrystalline cellulose	2	220
4	Sodium stearyl fumarate	5	550
5	Talc	3	330
6	Sodium saccharin	5	550
7	Mannitol	175	19250

**Table 4:** Weight variation specification as per IP.

Average weight of tablet	% Deviation
80 mg or less	±10
More than 80 mg but less than 250 mg	±7.5
250 mg or more	±5

**Table 5:** Evaluation parameters for the optimization of sodium starch glycolate.

Sr. no.	Evaluation parameters	F1 (1%)	F2 (2%)	F3 (4%)	F4 (6%)	F5 (8%)	F6 (10%)
1	Weight variation (IP)	Passed	Passed	Passed	Passed	Passed	Passed
2	Friability (%)	0.8	0.8	0.1	0.3	0.1	0.1
3	*Hardness (Kg/cm^2^) ± S.D	2.2 ± 0.57	1.6 ± 0.28	1.5 ± 0.28	1.5 ± 0.32	2.0 ± 0.57	1.8 ± 0.28
4	**Disintegration time (sec) ± S.D	80 ± 2.34	59 ± 6.67	**34 ± 2.63**	48 ± 6.38	78 ± 7.39	95 ± 6.97

*Average of three determinations.

**Average of six determinations.

**Table 6:** Evaluation parameters for the optimization of polyvinylpyrrolidone (PVP K-30) or microcrystalline cellulose as binder with optimized concentration of sodium starch glycolate.

Evaluation parameters	Weight variation (IP)	Friability (%)	*Hardness (Kg/cm^2^) ± S.D	**Disintegration time (sec) ± S.D
Formula no.				
F1	Passed	0.1	2.2 ± 0.28	60 ± 1.78
F2	Passed	0.2	1.8 ± 0.28	**45 ± 1.67**
F3	Passed	0.5	2.0 ± 0.00	69 ± 2.89
F4	Passed	0.3	3.2 ± 0.76	83 ± 2.40
F5	Passed	0.3	1.6 ± 0.50	90 ± 5.16
F6	Passed	0.8	2.5 ± 0.50	120 ± 5.77
F7	Passed	0.8	2.0 ± 0.00	145 ± 5.43
F8	Passed	0.1	1.5 ± 0.50	**36 ± 2.13**
F9	Passed	0.1	1.5 ± 0.28	47 ± 1.34
F10	Passed	0.2	1.5 ± 0.28	62 ± 1.10
F11	Passed	0.1	1.8 ± 0.28	75 ± 1.32
F12	Passed	0.1	1.5 ± 0.28	82 ± 2.08
F13	Passed	0.1	1.8 ± 0.28	96 ± 0.84
F14	Passed	0.1	1.8 ± 0.28	105 ± 0.73

*Average of three determinations.

**Average of six determinations.

**Table 7:** Official tests for salbutamol sulphate FDT.

Sr. no.	Evaluation parameters	Results
1	Weight variation (IP)	Passed
2	*Thickness (mm) ± S.D	3.63 ± 0.06
3	*Hardness (Kg/cm^2^) ± S.D	1.5 ± 0.29
4	Friability (%)	0.5
5	**Disintegration time (sec) ± S.D	45 ± 2.34
6	*Wetting time (sec) ± S.D	28 ± 1.53
7	*Drug content uniformity (mg)	103.6 ± 3.36

*Average of three determinations.

**Average of six determinations.

**Table 8:** Stability data of salbutamol sulphate FDT at room temperature and at ambient humidity.

Time interval	Data of three primary batches on								
	0 day			15th day			30th day		
Evaluation parameters	B-1	B-2	B-3	B-1	B-2	B-3	B-1	B-2	B-3
*Hardness (Kg/cm^2^) ± S.D	1.5 ± 0.29	1.8 ± 0.29	1.5 ± 0.29	1.5 ± 0.00	1.5 ± 0.00	1.7 ± 0.29	1.5 ± 0.00	1.5 ± 0.29	1.5 ± 0.29
Friability (%)	1	0.6	1	0.2	0.3	0.2	0.1	0.1	0.1
*Drug content uniformity (mg) ± S.D	100.8 ± 3.36	95.6 ± 2.34	93.8 ± 1.24	99.5 ± 2.14	94.5 ± 2.67	94.8 ± 1.23	98.3 ± 2.74	95.4 ± 2.36	95.7 ± 1.71
**Disintegration time (sec) ± S.D	39 ± 2.28	47 ± 1.80	42 ± 3.01	42 ± 3.97	50 ± 4.52	47 ± 1.66	46 ± 2.83	49 ± 2.52	48 ± 3.75

*Average of three determinations/batch.

**Average of six determinations/batch.

**Table 9:** Stability data of salbutamol sulphate FDT at temperature (40° ± 2°C) and at ambient humidity.

Time interval	Data of three primary batches on								
	0 day			15th day			30th day		
Evaluation parameters	B-1	B-2	B-3	B-1	B-2	B-3	B-1	B-2	B-3
*Hardness (Kg/cm^2^) ± S.D	1.5 ± 0.29	1.8 ± 0.29	1.5 ± 0.29	2.5 ± 0.00	2.2 ± 0.29	2.5 ± 0.00	2.5 ± 0.00	2.5 ± 0.29	3.2 ± 0.29
Friability (%)	1	0.6	1	0.1	0.2	0.9	0.6	0.5	0.1
*Drug content uniformity (mg) ± S.D	100.8 ± 3.36	95.6 ± 2.34	93.8 ± 1.24	98.5 ± 2.14	99.4 ± 2.67	91.42 ± 3.64	92.8 ± 1.98	99 ± 1.65	97.6 ± 3.63
**Disintegration time (sec) ± S.D	39 ± 2.28	47 ± 1.80	42 ± 3.01	49 ± 2.38	55 ± 3.08	51 ± 1.76	55 ± 2.09	61 ± 1.89	58 ± 2.96

*Average of three determinations/batch.

**Average of six determinations/batch.
